# Treatment with Modified Extracts of the Microalga *Planktochlorella nurekis* Attenuates the Development of Stress-Induced Senescence in Human Skin Cells

**DOI:** 10.3390/nu12041005

**Published:** 2020-04-06

**Authors:** Jagoda Adamczyk-Grochala, Maciej Wnuk, Magdalena Duda, Janusz Zuczek, Anna Lewinska

**Affiliations:** 1 Department of Biotechnology, University of Rzeszow, Pigonia 1, 35-310 Rzeszow, Poland; ag1jagoda@gmail.com (J.A.-G.); mawnuk@gmail.com (M.W.); 2 Bioorganic Technologies sp. z o.o., Sedziszow Malopolski, Sielec 1A, 39-120 Sielec, Poland; mduda@bioorganictechnologies.pl (M.D.); janusz@bioorganictechnologies.pl (J.Z.)

**Keywords:** microalgae, *Planktochlorella nurekis*, skin cells, proliferation, senescence

## Abstract

More recently, we have proposed a safe non-vector approach to modifying the biochemical profiles of the microalga *Planktochlorella nurekis* and obtained twelve clones with improved content of lipids and selected pigments and B vitamins and antioxidant activity compared to unaffected cells. In the present study, the biological activity of water and ethanolic extracts of modified clones is investigated in the context of their applications in the cosmetic industry and regenerative medicine. Extract-mediated effects on cell cycle progression, proliferation, migration, mitogenic response, apoptosis induction, and oxidative and nitrosative stress promotion were analyzed in normal human fibroblasts and keratinocytes in vitro. Microalgal extracts did not promote cell proliferation and were relatively non-cytotoxic when short-term treatment was considered. Long-term stimulation with selected microalgal extracts attenuated the development of oxidative stress-induced senescence in skin cells that, at least in part, was correlated with nitric oxide signaling and increased niacin and biotin levels compared to an unmodified microalgal clone. We postulate that selected microalgal extracts of *Planktochlorella nurekis* can be considered to be used in skin anti-aging therapy.

## 1. Introduction

The skin is a natural barrier that protects the human body against a number of environmental stressors, such as physical, chemical, or biological agents. Environmental factors such as sun radiation (ultraviolet radiation, visible light, and infra-red radiation), air pollutants (polycyclic aromatic hydrocarbons (PAH), volatile organic compounds (VOCs), particulate matters (PMs), and ozone) and tobacco smoke may affect and/or promote the progression of inflammatory skin diseases such as atopic dermatitis (AD), acne and psoriasis, skin aging, and cancerogenesis (e.g., photo-aging and photo-carcinogenesis) [1,2]. Moreover, the skin may be subjected to photo-pollution stress (combined action of UV radiation and air pollutants, such as PAH and PMs) that may result in synergistic photo-toxic damage [2,3].

Skin aging, a complex biological process that can be modulated by several intrinsic (chronological or intrinsic skin aging) and environmental factors (e.g., UV radiation, extrinsic skin aging), is accompanied by both phenotypic changes in cutaneous cells and structural and functional changes of extracellular matrix (ECM) components, namely collagens, elastin and proteoglycans that are needed to maintain the tensile strength, elasticity and hydration of the skin [4,5]. Mitochondrial DNA damage, increased reactive oxygen species (ROS) production, and telomere shortening are considered to be major players during intrinsic skin aging [4,5]. Moreover, the accumulation of replicative old senescent skin cells may also promote the pathological remodeling of ECM [4]. Cumulative exposure to UV radiation can result in the damage of chromophore-rich cutaneous biomolecules and oxidative stress, affecting skin cells and matrix components, and stimulate the expression of ECM proteases (e.g., matrix metalloproteinases, MMPs) via transcription factor activator protein-1 (AP-1) signaling, leading to collagen degradation and wrinkle formation [4].

Macroalgae (multicellular algae) and microalgae (unicellular algae) are a rich source of natural bioactive compounds and secondary metabolites with antioxidant, anti-inflammatory, anti-microbial and anticancer activities that may have some beneficial effects during skin anti-aging therapies, de-pigmentation, and acne, and chronic wound and melanoma treatments [6,7,8]. As algae are exposed to UV-radiation-mediated oxidative stress in their natural environments and adapted to cope with oxidative biomolecule damage by the means of elevated production of antioxidants and UV-absorbing compounds, algal extracts and isolated chemicals may provide similar UV protection as sunscreens currently used in the market [8]. Photo-protective activity of selected algal compounds, namely, shinorine, porphyra-334, palythene, eckstolonol, eckol, mycosporine-glycine, mycosporine methylamine-serine, sargachromenol, fucoxanthin, tetraprenyltoluquinol chromane meroterpenoid, scytonemin, and sargaquinoic acid, has been previously reported [7,9,10,11,12]. A number of algal bioactive compounds may protect against photo-aging by decreasing the levels of MMPs and maintaining the pools of skin collagen [6,7,8]. Extracts of the green microalga *Chlorella vulgaris* may also promote collagen synthesis in the skin, stimulate tissue regeneration and limit wrinkle formation, and thus, may have applications in skin anti-aging therapies [6,8].

Although algae and related groups are widely studied, only a limited number of microalgal and cyanobacterial species have been comprehensively characterized and commercially used, namely, *Spirulina*, *Chlorella*, *Haematococcus*, *Dunaliella*, *Botryococcus*, *Phaeodactylum,* and *Porphyridium* [8,13,14]. In 2014, a new microalgal species was reported, namely, *Planktochlorella nurekis* [15]. More recently, we provided a comprehensive biochemical characterization of the microalga *Planktochlorella nurekis* and selected twelve clones with elevated levels of lipids and several pigments and B vitamins, and increased antioxidant activity [16]. However, *Planktochlorella nurekis*-mediated effects in cellular models in vitro have never been addressed, e.g., the ability to stimulate cell proliferation and migration and to promote some anti-aging effects. In the present study, we use human fibroblasts and keratinocytes to investigate mitogenic, promigratory and anti-senescence activity of water and ethanolic extracts obtained from twelve modified clones of *Planktochlorella nurekis* and their corresponding unaffected cells. Extract-mediated ability to block the development of oxidative stress-induced senescence in human skin cells is documented and discussed.

## 2. Materials and Methods 

### 2.1. Cell Culture and Extract Preparation and Treatment

Human foreskin fibroblasts BJ (lot 62341989, catalog number ATCC^®^ CRL-2522^™^) were obtained from American Type Culture Collection (ATCC, Manassas, VA, USA) and human epidermal keratinocytes HEK (lot 3057, catalog number 102-05F and lot 1831898, catalog number C0015C) were obtained from Cell Application Inc. (San Diego, CA, USA) and Thermo Fisher Scientific (Waltham, MA, USA), respectively. Population doubling levels were monitored as described previously [17] and only replicative young cells with high proliferative potential were used. Cells were cultured at 37 °C in a cell culture incubator in the presence of 5% CO_2_. BJ fibroblasts were cultured in Dulbecco’s modified Eagle’s medium (DMEM) containing 10% fetal calf serum (FCS), 100 U/mL penicillin, 0.1 mg/mL streptomycin, and 0.25 μg/mL amphotericin B (Corning, Tewksbury, MA, USA). HEK cells were cultured in EpiLife^™^ basal medium with the Human Keratinocyte Growth Supplement Kit (HKGS Kit, Thermo Fisher Scientific, Waltham, MA, USA) containing bovine pituitary extract (BPE, 0.2% *v/v*), recombinant human insulin-like growth factor-I (0.01 µg/mL), hydrocortisone (0.18 µg/mL), bovine transferrin (5 µg/mL), human epidermal growth factor (0.2 ng/mL) and gentamicin/amphotericin. BJ cells were passaged using 0.25% trypsin/2.21 mM EDTA (Corning, Tewksbury, MA, USA) and HEK cells were passaged using 0.025% trypsin/0.01% EDTA (Thermo Fisher Scientific, Waltham, MA, USA) and trypsin was then inactivated using Trypsin Neutralizer Solution (Thermo Fisher Scientific, Waltham, MA, USA). Typically, cells were seeded at 10^4^ cells per cm^2^ of a culture flask. 

Twelve clones of the microalga *Planktochlorella nurekis* with improved biochemical features were used [16]. A detailed description of biochemical profiles of modified microalgal clones can be found elsewhere [16]. To prepare microalgal extracts of twelve clones of the microalga *Planktochlorella nurekis* and one control clone, two solvents were considered, namely, water and 80% ethanol. To obtain water extracts (WE), 100 mg of microalgal dry weight were added to sterile ultra pure water to give the stock concentration of 100 mg/mL. The samples were then boiled at 100 °C for 10 min and centrifuged (13,000 rpm, RT, 10 min). Supernatants were collected and stored until use at −20 °C. To obtain ethanolic extracts (EE), 100 mg of microalgal dry weight were added to 80% ethanol to give the stock concentration of 100 mg/mL. The samples were then incubated at 37 °C for 24 h with shaking (1000 rpm) and centrifuged (13,000 rpm, RT, 10 min). Supernatants were collected and stored until use at −20 °C. Fibroblasts and keratinocytes were treated with microalgal extracts for 24 h (the majority of experiments), up to 72 h (wound healing assay) or up to 7 days (senescence-associated beta-galactosidase activity). The solvent action (water, 80% ethanol) alone was also considered and the solvents used had no effect on cells.

### 2.2. MTT Assay

To study the extract-mediated changes in metabolic activity (thiazolyl blue tetrazolium bromide (MTT) assay), BJ and HEK cells were seeded at the concentration of 5000 cells per a well of a 96-well plate and cultured overnight. Microalgal extracts were then added (water extracts at the concentrations ranging from 1 ng/mL to 1000 µg/mL and ethanolic extracts at the concentrations ranging from 1 ng/mL to 500 µg/mL) for 24 h. After the removal of microalgal extracts, cells were incubated with MTT solution (0.5 mg/mL, 4 h). After incubation, the MTT solution was removed, and 200 µL DMSO was added to dissolve the formazan crystals. Absorbance was read at 570 and 630 nm using a microplate reader. Metabolic activity was calculated as a ΔA (A570-A630). Metabolic activity at control conditions (untreated cells) was considered as 100%. According to MTT results, the concentrations of 100 µg/mL water and 100 µg/mL ethanolic extracts and the concentrations of 100 µg/mL water and 1 µg/mL ethanolic extracts were selected for further analysis (BJ and HEK cells, respectively). 

### 2.3. Cell Cycle

Extract-mediated changes in the phases of the cell cycle were analyzed using a Muse^®^ Cell Analyzer and a Muse^®^ Cell Cycle Assay Kit according to the manufacturer’s instructions (Merck KGaA, Darmstadt, Germany). Briefly, BJ and HEK cells were treated with microalgal extracts for 24 h and then fixed using ice-cold 70% ethanol at −20 °C. Cells were then stained using kit reagent and G0/G1 (gap 1), S (synthesis), and G2 (gap 2)/M (mitosis) phases were analyzed [%]. Representative histograms are also shown.

### 2.4. Cell Proliferation

Extract-mediated changes in cell proliferation were investigated as cell number analysis and Ki67 (proliferation marker) immunostaining. The cell number was automatically calculated using TC10^™^ Automated Cell Counter (Bio-Rad, Hercules, CA, USA). The subpopulations of Ki67-positive and Ki67-negative cells (%) were analyzed using a Muse^®^ Cell Analyzer and a Muse^®^ Ki67 Proliferation Kit (Merck KGaA, Darmstadt, Germany). Briefly, BJ and HEK cells were treated with microalgal extracts for 24 h and then fixed and permeabilized using kit reagents for fixation and permeabilization and then incubated with the Ki67 antibody. Control Ki67-non-specific antibody was used to calculate the subpopulation of Ki67-negative cells (grey histogram). Representative histograms are also shown.

### 2.5. Mitogenic Activity 

Extract-induced mitogenic activity was revealed using a Muse^®^ Cell Analyzer and a Muse^®^ MAPK Activation Dual Detection Kit. Briefly, BJ and HEK cells were treated with microalgal extracts for 24 h, fixed, permeabilized and extracellular signal-regulated kinase 1/2 (ERK1/2) activity was measured using two directly conjugated antibodies, a phospho-specific anti-phospho-ERK1/2 (Thr202/Tyr204, Thr185/Tyr187)-phycoerythrin, and anti-ERK1/2-PECy5 conjugated antibody (Merck KGaA, Darmstadt, Germany) according to the manufacturer’s instructions. Three subpopulations were considered, namely, ERK1/2-positive (inactivated fraction), phospho-ERK1/2-positive (activated fraction), and ERK1/2-negative (non-expressing fraction). Representative dot-plots are also shown.

### 2.6. Wound Healing Assay

Extract-mediated cell migration was evaluated using a wound healing assay [18]. Briefly, BJ and HEK cells were cultured until almost 100% confluency was achieved, and a fresh medium containing 100 μg/mL water extracts was added. A sterile pipette tip was applied to make a cross-shaped wound by streaking across a monolayer of BJ and HEK cells. The migration of cells into the wound was documented at time 0, 24, 48, and 72 h after wounding using an inverted microscope. Every 24 h, a fresh medium containing 100 μg/mL water extract was added.

### 2.7. Apoptosis versus Necrosis

Extract-induced apoptosis was evaluated using a Muse^®^ Cell Analyzer and a Muse^®^ Annexin V and Dead Cell Assay Kit (Merck KGaA, Darmstadt, Germany). Briefly, BJ and HEK cells were stained according to manufacturer’s instructions and four-cell subpopulations were revealed, namely, (1) non-apoptotic cells (live cells): Annexin V (−) and 7-AAD (−), (2) early apoptotic cells: Annexin V (+) and 7-AAD (−), (3) late apoptotic and dead cells: Annexin V (+) and 7-AAD (+), and (4) mostly nuclear debris (necrotic cells): Annexin V (−) and 7-AAD (+) (%). Representative dot-plots are also shown. Necrosis was also evaluated using a trypan blue exclusion assay. BJ and HEK cells were incubated with 0.4% trypan blue, and then dead cells with porous cell membranes (blue-stained cells) were automatically calculated (%) using a TC10™ Automated Cell Counter (Bio-Rad, Hercules, CA, USA).

### 2.8. Superoxide and Nitric Oxide Levels

Extract-mediated changes in the levels of superoxide and nitric oxide were evaluated using a Muse^®^ Cell Analyzer and a Muse^®^ Oxidative Stress Kit and Muse^®^ Nitric Oxide Kit, respectively, according to the manufacturer’s instructions. Briefly, for evaluation of oxidative stress, BJ and HEK cells were incubated with superoxide specific fluorogenic probe dihydroethidium, and, for evaluation of nitrosative stress, human fibroblasts and keratinocytes were incubated with nitric oxide specific fluorogenic probe DAX-J2^™^ Orange and a death marker, 7-AAD. Two subpopulations were revealed, namely, superoxide-positive and superoxide-negative and four subpopulations were documented, namely, (1) nitric oxide-negative, 7-AAD-negative, (2) nitric oxide-positive, 7-AAD-negative, (3) nitric oxide-positive, 7-AAD-positive, and (4) nitric oxide-negative, 7-AAD-positive, respectively. Representative histograms or dot-plots are also shown.

### 2.9. Senescence-Associated Beta-Galactosidase Activity

A cellular model of hydrogen peroxide-induced senescence was considered, as described previously [17,19]. Briefly, BJ and HEK cells were treated with 100 µM hydrogen peroxide for 2 h, and then water and ethanolic extracts were added for up to 7 days to analyze their protective effects. Every 48 h, a fresh medium containing microalgal extracts was added. After 7 days of hydrogen peroxide removal, senescence-associated beta-galactosidase (SA-beta-gal) activity was assayed as described previously [17,19]. The effect of water and ethanolic extracts alone (pro-senescence activity) was also investigated. BJ and HEK cells were treated with water and ethanolic extracts for 24 h, and, 7 days after microalgal extracts removal, SA-beta-gal activity was analyzed. SA-beta-gal-positive and SA-beta-gal-negative cells were scored (%).

### 2.10. Preliminary Analysis of Anticancer Activity

Water and ethanolic extracts were also initially analyzed in terms of their anticancer activity. Extract-mediated changes in metabolic activity (MTT assay) of normal human fibroblasts (BJ cells) were compared with extract-induced changes in metabolic activity of selected human cancer cells, namely, MDA-MB-231 breast cancer cells (ATCC^®^ HTB-26^™^, ATCC, Manassas, VA, USA), U-2 OS osteosarcoma cells (92022711, ECACC, Porton Down, Salisbury, UK), and U-251 MG glioblastoma cells (09063001, ECACC, Porton Down, Salisbury, UK). Briefly, cells were seeded at 5000 cells per well of a 96-well plate and cultured overnight. Cells were then treated with water and ethanolic extracts (100 µg/mL) for 24 h, and, after microalgal extracts removal, cells were incubated with MTT reagent as described above. 

### 2.11. Statistical Analysis

The results represent the mean ± standard deviation (SD) from at least three independent experiments. Statistical significance was assessed by 1-way analysis of variance (ANOVA) using GraphPad Prism 5 and with Dunnett’s multiple comparison test.

## 3. Results and Discussion

### 3.1. The Effect of Water and Ethanolic Extracts of the Microalga Planktochlorella Nurekis on Cell Cycle Progression and Proliferation of Human Skin Cells

In 2014, a new species of microalgae was reported, namely, *Planktochlorella nurekis* [15]. More recently, we comprehensively characterized its biochemical features and also selected twelve microalgal clones with improved biochemical profiles based on colchicine and cytochalasin B-mediated changes in ploidy state and DNA content [16]. However, the biological activity of water and ethanolic extracts of the microalga *Planktochlorella nurekis* has not been addressed. In the present study, we focus on the evaluation of extract-mediated effects on cell proliferation, migration, mitogenic activity, overall cytotoxicity and the ability to attenuate the development of oxidative stress-induced senescence in human skin cells that may have potential applications in the cosmetic industry and regenerative medicine. We also asked if the modified and improved clones of the microalga *Planktochlorella nurekis* may have some advantages over control unaffected microalgal cells.

**Figure 1:**
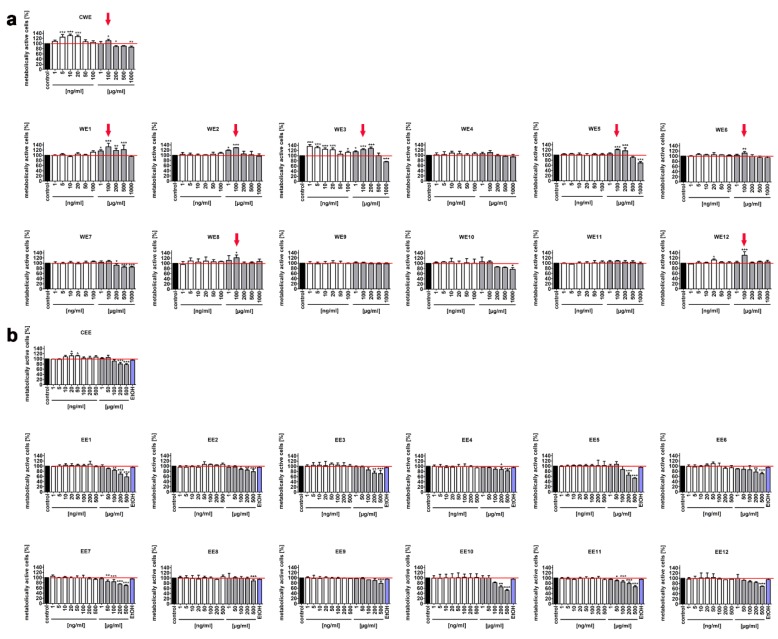
Extract-mediated changes in metabolic activity (MTT assay) of normal human fibroblasts (BJ). Metabolic activity at standard growth conditions (control, a black bar) is considered as 100%. (**a**) The effects of water extracts (WE, twelve modified clones from WE1 to WE12) are shown. Control clone water extract is denoted as CWE. (**b**) The effects of ethanolic extracts (EE, twelve modified clones from EE1 to EE12) are shown. Control clone ethanolic extract is denoted as CEE. To emphasize extract action, a red horizontal line is added. The effect of 80% ethanol (a solvent of ethanolic extracts, a violet bar) is also shown. White bars indicate the extract concentrations in ng/mL, whereas grey bars indicate the extract concentrations in µg/mL. Red arrows indicate the concentration of water extract (100 µg/mL) that was selected for further analysis based on the stimulatory effect on the metabolic activity of the majority of modified clone water extracts used. Bars indicate SD, *n* = 5, *** *p* < 0.001, ** *p* < 0.01, * *p* < 0.05 compared to the control (ANOVA and Dunnett’s a posteriori test).

Two types of skin cells were selected for the analysis, namely, normal human fibroblasts (BJ cells) and normal human keratinocytes (HEK cells). As extracts of different *Chlorella* species may act on human and rodent cells at a wide range of concentrations and the effects may also rely on solvents used [20,21,22,23,24], we decided to study both water and ethanolic extracts and the concentrations ranging from 1 to 1000 µg/mL (Figure 1).

We noticed that 100 µg/mL water extracts of seven modified clones (WE1, WE2, WE3, WE5, WE6, WE8, WE12) promoted metabolic activity (MTT assay) of BJ cells compared to control conditions (Figure 1a, red arrows). Control water extract (CWE) also caused a slight increase in metabolic activity (Figure 1a, a red arrow). A similar effect was not observed when BJ cells were treated with ethanolic extracts (Figure 1b). Moreover, we have established that ethanolic extracts can be applied at the concentrations up to 100 µg/mL without promoting adverse changes in metabolic activity (Figure 1b). At the concentration of 500 µg/mL, all ethanolic extracts, both modified and control, caused a statistically significant decrease in metabolic activity of BJ cells (Figure 1b, *p* < 0.001).

More recently, it has been observed that a hot water extract of *Chlorella vulgaris* at the concentrations of 400 and 600 µg/mL increased the number of viable cells of young human dermal fibroblasts (HDFs) [24]. Interestingly, the extract-mediated effect depended on replicative age of HDFs, namely, stimulatory effects in pre-senescent and senescent HDFs were noticed when 200 and 100 µg/mL water extract was used, respectively, and the concentrations ranging from 500 to 800 µg/mL decreased cell viability of senescent HDFs [24]. Water and 80% ethanolic extracts of *Chlorella sorokiniana* did not affect the cell viability of Chinese hamster lung fibroblasts (V79 cells) when used at the concentrations up to 80 and 20 µg/mL, respectively [23]. However, treatment with 12.5, 25, and 50 μg/mL hot water extract of *Chlorella vulgaris* increased cell viability by 124%, 135.4%, and 155.0% in IEC-6 rat small intestine epithelial cells compared to untreated cells [21]. A slight cytotoxic effect of 50 and 100 μg/mL ethanolic extract of *Chlorella vulgaris* was also reported using 3T3-L1 mouse preadipocytes as a cellular model in vitro [22].

**Figure 2:**
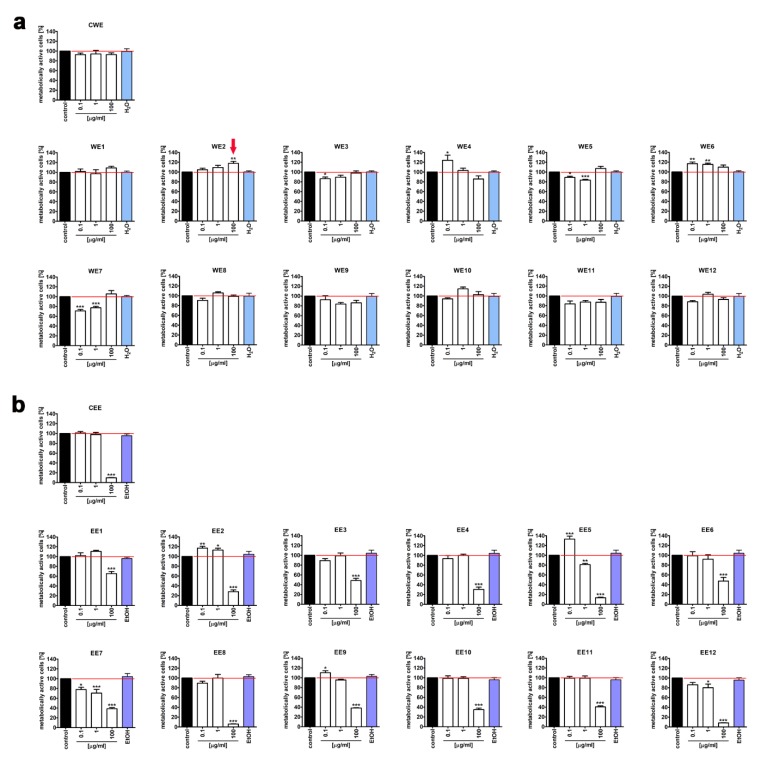
Extract-mediated changes in metabolic activity (MTT assay) of normal human keratinocytes (HEK). Metabolic activity at standard growth conditions (control, a black bar) is considered as 100%. (**a**) The effects of water extracts (WE, twelve modified clones from WE1 to WE12, white bars) are shown. Control clone water extract is denoted as CWE. (**b**) The effects of ethanolic extracts (EE, twelve modified clones from EE1 to EE12, white bars) are shown. Control clone ethanolic extract is denoted as CEE. To emphasize extract action, a red horizontal line is added. The effect of water (a blue bar) and 80% ethanol (a violet bar) (solvents used) is also shown. Red arrow indicates the concentration of water extract (100 µg/mL) that was selected for further analysis based on the stimulatory effect on the metabolic activity of BJ cells of the majority of modified clone water extracts used. Bars indicate SD, *n* = 5, *** *p* < 0.001, ** *p* < 0.01, * *p* < 0.05 compared to the control (ANOVA and Dunnett’s a posteriori test).

Three selected concentrations (0.1, 1, and 100 µg/mL) were also considered when studying extract-mediated changes in the metabolic activity of human keratinocytes (Figure 2). In general, HEK cells were found to be more sensitive to microalgal extract treatment than BJ cells, especially in terms of ethanolic extract at the concentration of 100 µg/mL (Figure 1 and Figure 2b). Moreover, a pro-stimulatory effect on the metabolic activity of HEK cells was limited to 100 µg/mL WE2 (Figure 2a, a red arrow). There are no literature data on the effects of *Chlorella* sp. extracts on normal keratinocytes in vitro. The effect of two extracts obtained from two different microalgal species derived from the Blue Lagoon, namely coccoid and filamentous algae, on the vitality of primary human epidermal keratinocytes was studied [25]. Coccoid algae extract (157 µg/mL, *p* < 0.05) promoted cell viability, whereas filamentous algae extract, when used from the concentration of 12 µg/mL, stimulated cytotoxic effect (*p* < 0.05) [25]. However, the authors did not provide information about the microalgal species used [25]. It has also been reported that 0.1% (1 mg/mL) aqueous crude extract of the cyanobacterium *Spirulina platensis* caused a 2-fold increase in cell viability of the HS2 human keratinocyte cell line [26].

Based on obtained results, 100 µg/mL water extracts (from WE1 to WE12 and control CWE) and 100 µg/mL ethanolic extracts (from EE1 to EE12 and control CEE) were selected for further analysis using BJ cells, and 100 µg/mL water extracts (from WE1 to WE12 and control CWE) and 1 µg/mL ethanolic extract (from EE1 to EE12 and control CEE) were selected for further analysis using HEK cells. As the MTT test measures direct change in metabolic activity (the activity of mitochondrial dehydrogenases), one cannot discriminate between extract-mediated changes in cell proliferation, cell number, or cytostatic or cytotoxic effects.

**Figure 3:**
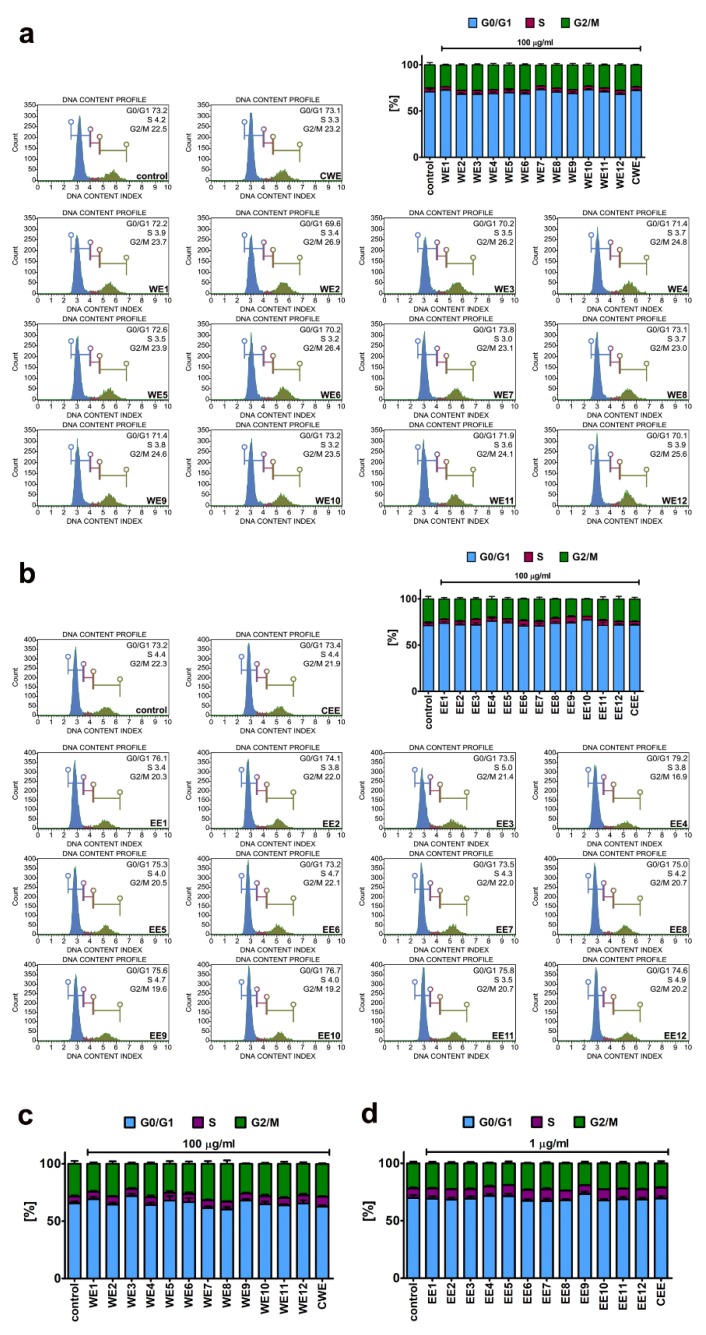
Extract-mediated changes in the cell cycle of BJ cells ((**a**), 100 µg/mL water extracts; (**b**), 100 µg/mL ethanolic extracts) and HEK cells ((**c**), 100 µg/mL water extracts; (**d**), 1 µg/mL ethanolic extracts). (**a**,**c**) The effects of water extracts (WE, twelve modified clones from WE1 to WE12) are shown. Control clone water extract is denoted as CWE. (**b**,**d**) The effects of ethanolic extracts (EE, twelve modified clones from EE1 to EE12) are shown. Control clone ethanolic extract is denoted as CEE. The percentage of cells in the G0/G1, S, and G2/M phases of the cell cycle was assessed using a Muse^®^ Cell Analyzer and a Muse^®^ Cell Cycle Kit. Representative histograms are shown (BJ cells). Bars indicate SD, *n* = 3.

**Figure 4:**
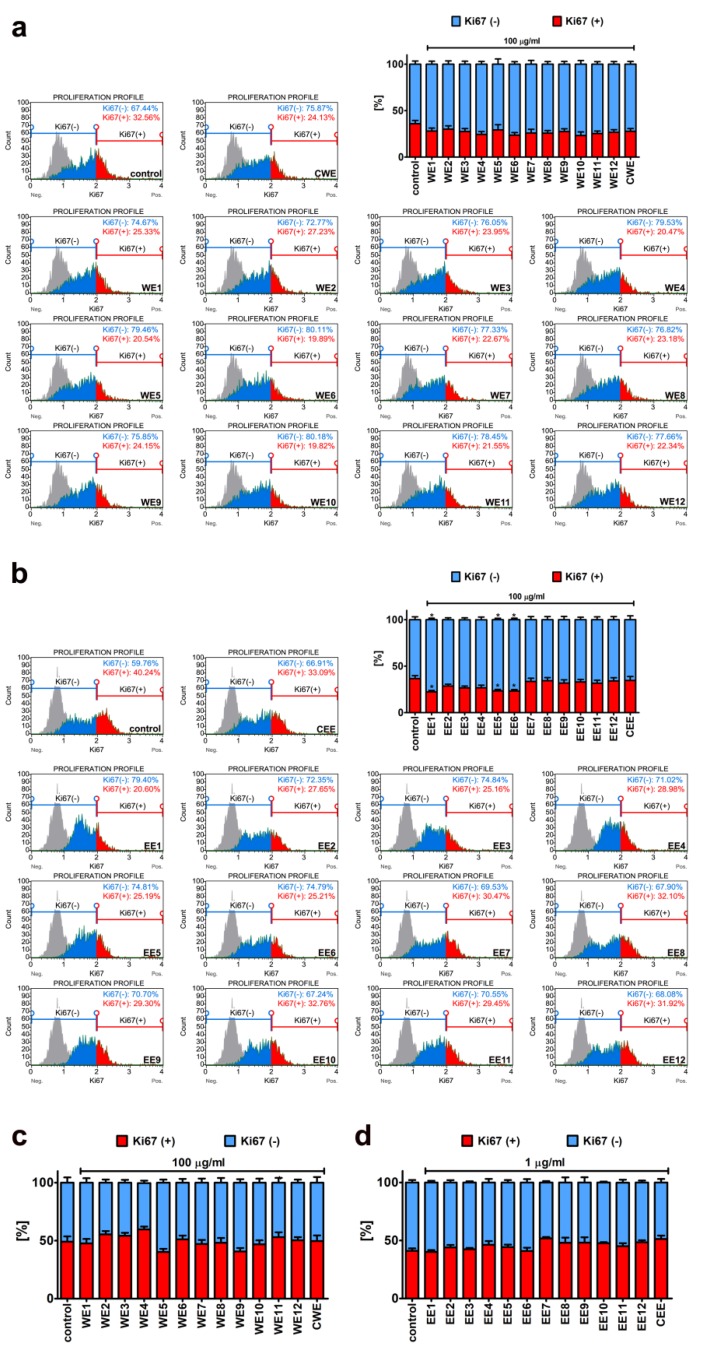
Extract-mediated changes in cell proliferation of BJ cells ((**a**), 100 µg/mL water extracts; (**b**), 100 µg/mL ethanolic extracts) and HEK cells ((**c**), 100 µg/mL water extracts; (**d**), 1 µg/mL ethanolic extracts). (**a**,**c**) The effects of water extracts (WE, twelve modified clones from WE1 to WE12) are shown. Control clone water extract is denoted as CWE. (**b**,**d**) The effects of ethanolic extracts (EE, twelve modified clones from EE1 to EE12) are shown. Control clone ethanolic extract is denoted as CEE. Cell proliferation was assayed using a Muse^®^ Cell Analyzer and a Muse^®^ Ki67 Proliferation Kit. Representative histograms are presented (BJ cells). A negative control without incubation with Ki67 specific antibody is denoted as a grey histogram in each analyzed sample. Bars indicate SD, *n* = 3. * *p* < 0.05 compared to the control (ANOVA and Dunnett’s a posteriori test).

**Figure 5:**
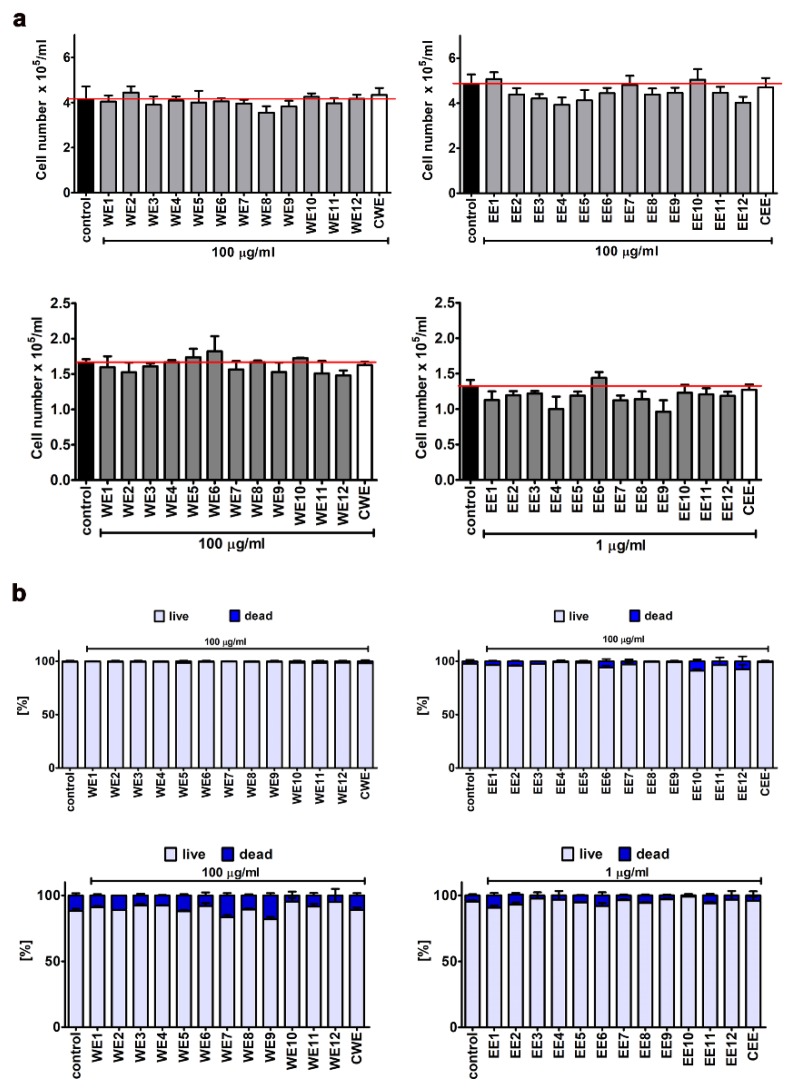
Extract-mediated changes in BJ and HEK cell numbers (**a**) and the levels of necrotic cells (trypan blue exclusion assay) (**b**). (**a**) Cell number was analyzed using TC10™ automated cell counter. To emphasize extract action, a red horizontal line is added. Black bars indicate control conditions, white bars indicate treatments with control extracts (CWE or CEE) and grey bars indicate treatments with modified water or ethanolic extracts. (**b**) BJ and HEK cells were incubated with 0.4% trypan blue and dead cells with porous cell membranes (blue-stained cells) were automatically calculated (%) using TC10™ automated cell counter. (**a**,**b**) Upper panel: BJ cells; lower panel: HEK cells. The effects of water extracts (WE, twelve modified clones from WE1 to WE12) and ethanolic extracts (EE, twelve modified clones from EE1 to EE12) are shown. Control clone water extract is denoted as CWE and control clone ethanolic extract is denoted as CEE. Bars indicate SD, *n* = 3.

Thus, we then decided to study extract-mediated pro-stimulatory effects more comprehensively (Figure 3, Figure 4 and Figure 5). Firstly, we analyzed extract-mediated changes in the phases of cell cycle of both BJ cells and HEK cells (Figure 3). No significant changes in the cell cycle progression were revealed upon stimulation of BJ and HEK cells with water and ethanolic extracts (Figure 3). It has been previously reported that the hot water extract of *Chlorella vulgaris* promoted the accumulation of cells at S and G2/M phases of the cell cycle in young human dermal fibroblasts [27]. However, the authors considered a four-times higher extract concentration (400 µg/mL) than used in the present study (100 µg/mL) [27]. Secondly, two cell proliferation parameters were considered, namely, Ki67 immunostaining (Figure 4) and cell number count (Figure 5a). In general, no significant changes in the levels of Ki67-positive cells were revealed after treatments with water and ethanolic extracts in HEK cells (Figure 4c,d). Ethanolic extracts EE1, EE5, and EE6 caused a slight decrease in the levels of Ki67-positive cells in BJ cells (Figure 4b, *p* < 0.05). Microalgal extracts also did not affect the cell number of BJ and HEK cells (Figure 5a).

Thus, one can conclude that extract-mediated stimulatory effects on metabolic activity (MTT assay) in BJ and HEK cells were not associated with increased cell proliferation (this study). In contrast, stimulation of IEC-6 rat small intestine epithelial cells with hot water extract of *Chlorella vulgaris* (up to 50 µg/mL) resulted in both an increase in metabolic activity and cell proliferation that was mediated by the activation of MAPK (ERK1/2) and PI3K/Akt pathways, pivotal regulators of cell survival, proliferation and growth [21]. Moreover, *Spirulina* crude protein also promoted the proliferation and migration of IEC-6 cells by activating the EGFR/MAPK signaling pathway [28]. Thus, we have then asked a question of whether microalgal extracts may modulate mitogenic response and cell migration of BJ cells and HEK cells. The mitogenic response was studied by analyzing the changes in the phosphorylation status of two mitogen-activated protein kinases, namely ERK1/2 (Figure S1). However, no ERK1/2 activation was revealed upon microalgal extract stimulation both in BJ cells and HEK cells (Figure S1). Finally, we have studied the effect of microalgal extracts on cell migration using wound healing assay (Figure S2). No extract-mediated stimulatory effects were observed in BJ cells (Figure S2a). In contrast, water extracts WE2, WE6, WE7, WE8, WE9, WE10, and WE11 promoted cell migration upon 48 h treatment after wounding in HEK cells (Figure S2b). There is a limited number of studies reporting the promigratory and wound healing effects of *Chlorella* sp. extracts in vitro and in vivo [29,30,31]. *Chlorella vulgaris* dressing promoted wound closure and minimized the formation of scar tissue during the healing process compared to the control wounds created on the dorsal surface of rats [29]. Moreover, oral (500 mg/kg) and topical (10%) administration of ROQUETTE *Chlorella* sp. had beneficial effects on skin lesions in mice [30]. The wound healing potential of *Arthrospira* (*Spirulina*) *platensis* extract has also been compared to the wound healing potential of *Chlorella vulgaris* extract using human fibroblasts as a cellular model [31]. It has been concluded that *Arthrospira* extract stimulated wound closure more efficiently than *Chlorella* extract [31]. The effect of solvent used has been also analyzed [32]. Treatment with 50 µg/mL water extract of *Spirulina platensis* promoted cell proliferation and migration of human fibroblasts in vitro, whereas the effects of methanolic and ethanolic extracts, when used at the same concentration, were mild to moderate [32]. The authors claimed that the promigratory and wound healing effects of water extracts were achieved by the presence of cinnamic acid, naringenin, kaempferol, temsirolimus, phosphatidylserine isomeric derivatives, and sulphoquinovosyl diacylglycerol [32]. Moreover, skin cream containing 1.125% *Spirulina platensis* crude extract showed an enhanced wound healing effect compared to control formulation using HS2 human keratinocytes as a cellular model [26].

### 3.2. Extract-Mediated Cytotoxicity, Oxidative and Nitrosative Stress

**Figure 6:**
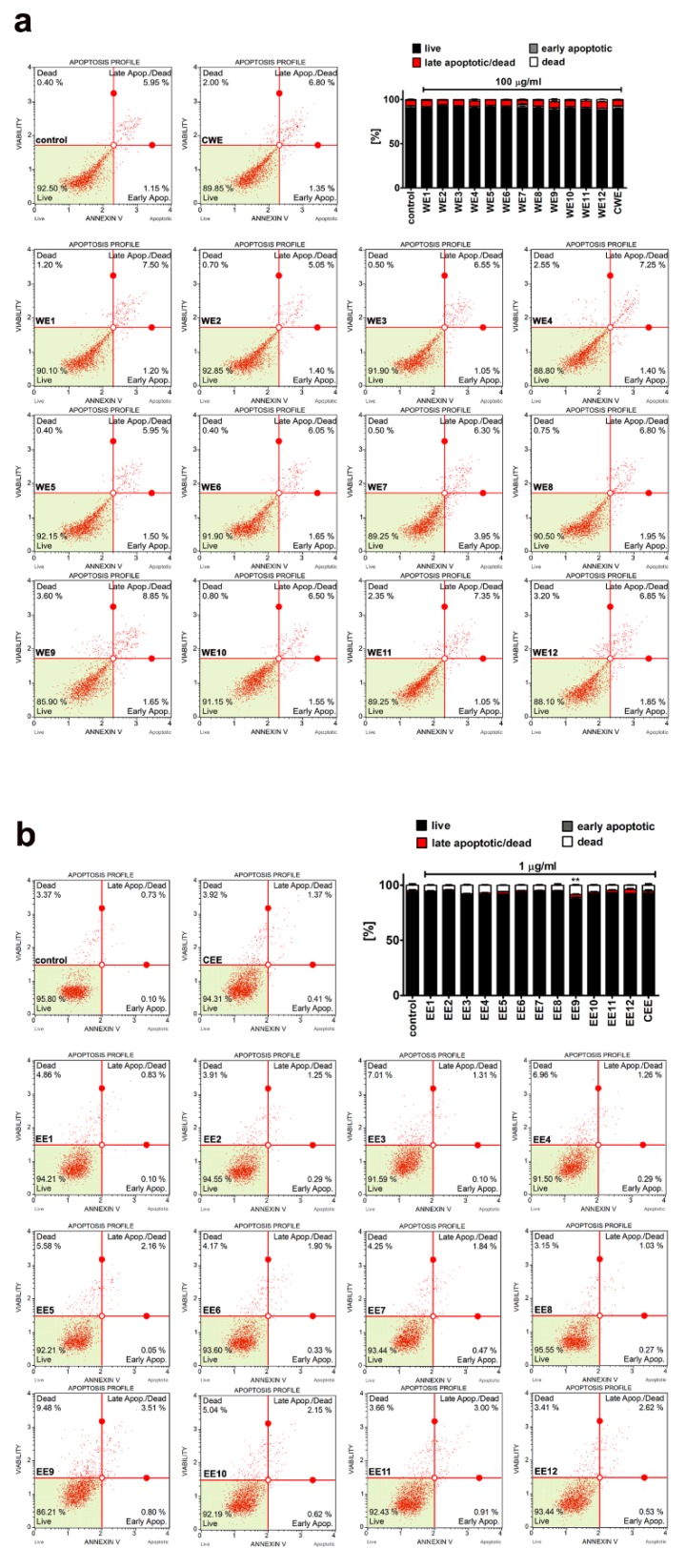
Extract-induced apoptosis in BJ cells ((**a**) 100 µg/mL water extracts) and HEK cells ((**b**) 1 µg/mL ethanolic extracts). (**a**) The effects of water extracts (WE, twelve modified clones from WE1 to WE12) are shown. Control clone water extract is denoted as CWE. (**b**) The effects of ethanolic extracts (EE, twelve modified clones from EE1 to EE12) are shown. Control clone ethanolic extract is denoted as CEE. Phosphatidylserine externalization was analyzed using a Muse^®^ Cell Analyzer and a Muse^®^ Annexin V and Dead Cell Assay Kit. Representative dot-plots are shown. Bars indicate SD, *n* = 3. ** *p* < 0.01 compared to the control (ANOVA and Dunnett’s a posteriori test).

**Figure 7:**
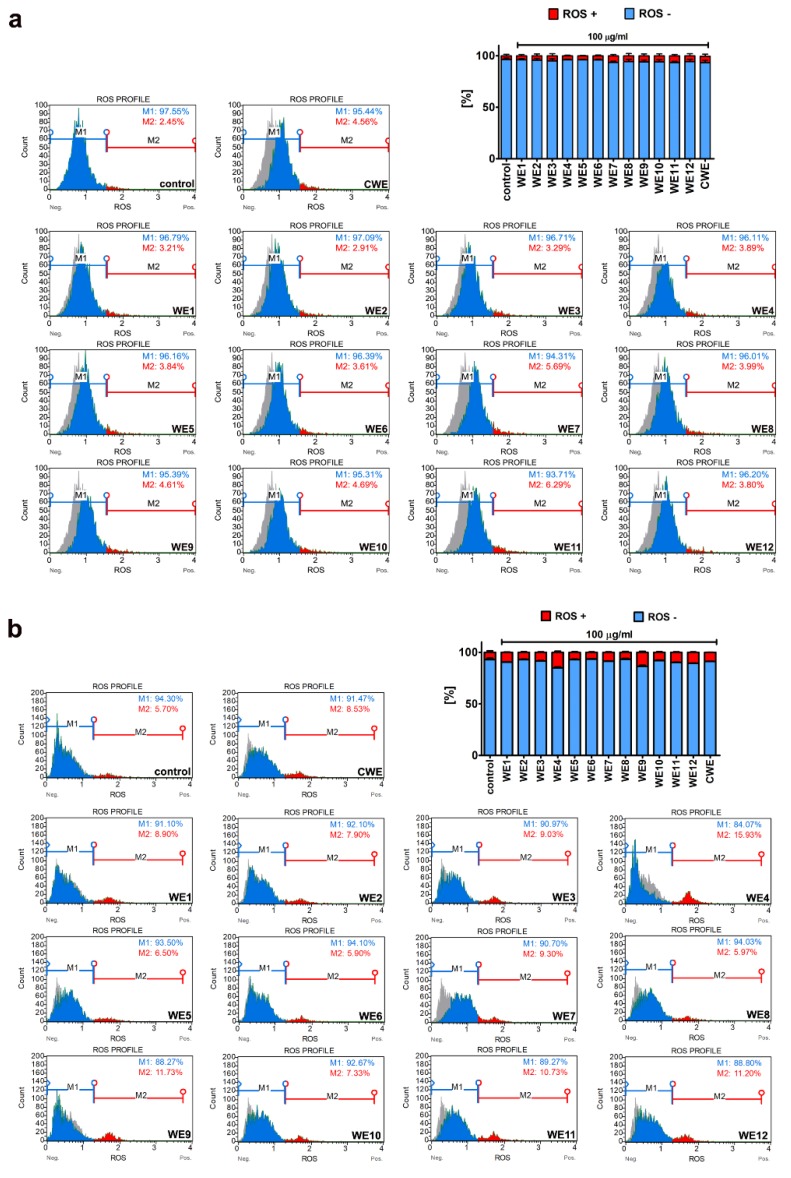
Extract-mediated changes in the levels of superoxide in BJ cells ((**a**) 100 µg/mL water extracts) and HEK cells ((**b**) 100 µg/mL water extracts). (**a**,**b**) The effects of water extracts (WE, twelve modified clones from WE1 to WE12) are shown. Control clone water extract is denoted as CWE. Superoxide levels were measured using a Muse^®^ Cell Analyzer and a Muse^®^ Oxidative Stress Kit. Representative histograms are presented. The control sample is denoted as a grey histogram in each sample analyzed. Bars indicate SD, *n* = 3.

**Figure 8:**
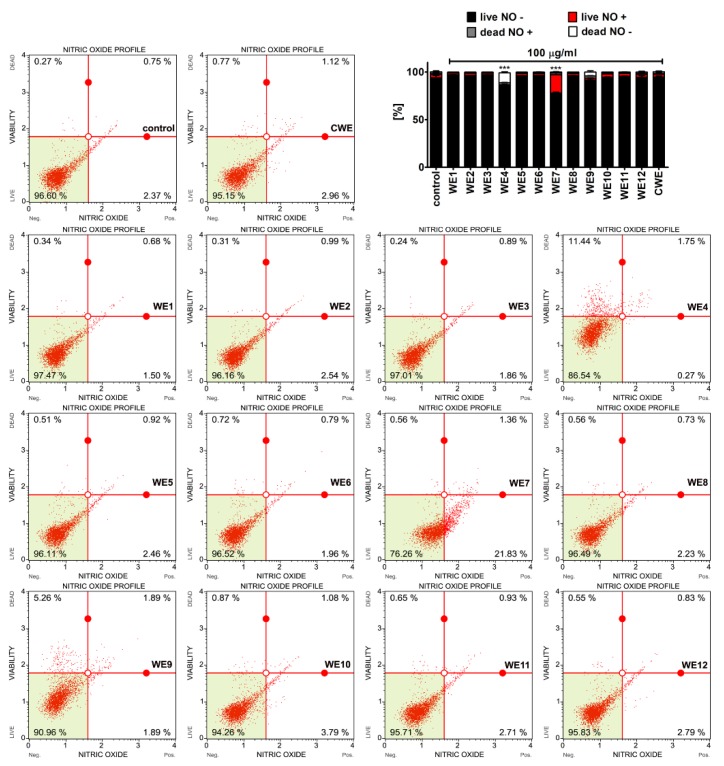
Extract-mediated changes in the levels of nitric oxide in BJ cells (100 µg/mL water extracts). The effects of water extracts (WE, twelve modified clones from WE1 to WE12) are shown. Control clone water extract is denoted as CWE. Nitric oxide levels were measured using a Muse^®^ Cell Analyzer and a Muse^®^ Nitric Oxide Kit. Representative dot-plots are presented. Bars indicate SD, *n* = 3. *** *p* < 0.001 compared to the control (ANOVA and Dunnett’s a posteriori test).

We have then considered if water and ethanolic extracts may also exert some adverse effects, namely, extract-mediated cytotoxicity, oxidative, and nitrosative stress were investigated (Figure 5b, Figure 6, Figure 7 and Figure 8).

No significant increase in the levels of necrotic cells was noticed upon microalgal extract stimulation in BJ cells and HEK cells, as judged by trypan blue exclusion assay (Figure 5b). However, a very slight increase in necrotic cell fraction in HEK cells was observed after treatment with ethanolic extract EE9, as revealed using a 7-AAD death marker (Figure 6b, *p* < 0.01). In contrast, no signs of an apoptotic mode of cell death were documented after treatment with both water and ethanolic extracts in BJ and HEK cells (Figure 6).

Thus, one can conclude that selected and analyzed concentrations of microalgal extracts were relatively non-cytotoxic when short-term treatment (24 h) was considered (this study). It has also been reported that a water extract of *Chlorella vulgaris* (400 µg/mL) lowered the levels of early apoptotic cells compared to control conditions in young human fibroblasts [27]. However, the levels of apoptotic cells at standard growth conditions were established to be more than 35%, which suggest the effect of some stress stimuli [27]. The solvent used and type of cells considered may also account for the cytotoxicity results. Briefly, 100 and 250 µg/mL methanolic extract of *Chlorella* elevated the levels of late apoptotic cells to 60.25% and 82.66% compared to untreated 3T3-L1 preadipocytes (25.88% of late apoptotic cells), respectively [20]. 

It is widely accepted that oxidative stress and nitrosative stress-promoting conditions can be detrimental to the cellular components causing, e.g., DNA and protein oxidative damage [33]. However, slight to moderate changes in the levels of reactive oxygen species (ROS) and reactive nitrogen species (RNS) may also have regulatory roles in the cell being a part of signal transduction pathways [33,34,35,36]. Thus, we then decided to analyze the effect of microalgal extracts on the levels of superoxide (Figure 7) and nitric oxide (Figure 8).

Microalgal extracts did not induce oxidative stress in BJ cells (Figure 7a). In HEK cells, a minor increase in the levels of superoxide was observed after treatment with water extracts WE4 and WE9 (Figure 7b). However, these effects were of no statistical significance (Figure 7b). Except for a statistically significant increase in the levels of nitric oxide of about 20% in WE7-treated BJ cells compared to control conditions (Figure 8, *p* < 0.001), microalgal extracts did not stimulate the production of nitric oxide in BJ cells (Figure 8) and HEK cells (data not shown). This moderate increase in the levels of nitric oxide after WE7 treatment may have a regulatory and signal transduction role. Indeed, the protective function of nitric oxide against UVB radiation-mediated oxidative stress and copper toxicity has been established in different *Chlorella* species [37,38]. The addition of nitric oxide to *Chlorella pyrenoidosa* suspensions irradiated by UVB promoted the activity of catalase and superoxide dismutase [37]. Moreover, exogenous nitric oxide limited copper-induced oxidative burst in *Chlorella vulgaris* [38]. Thus, nitric oxide may have an antioxidative role, at least in part, during oxidative stress conditions [37,38].

### 3.3. Attenuation of the Development of Stress-Induced Senescence in Skin Cells by Microalgal Extracts

**Figure 9:**
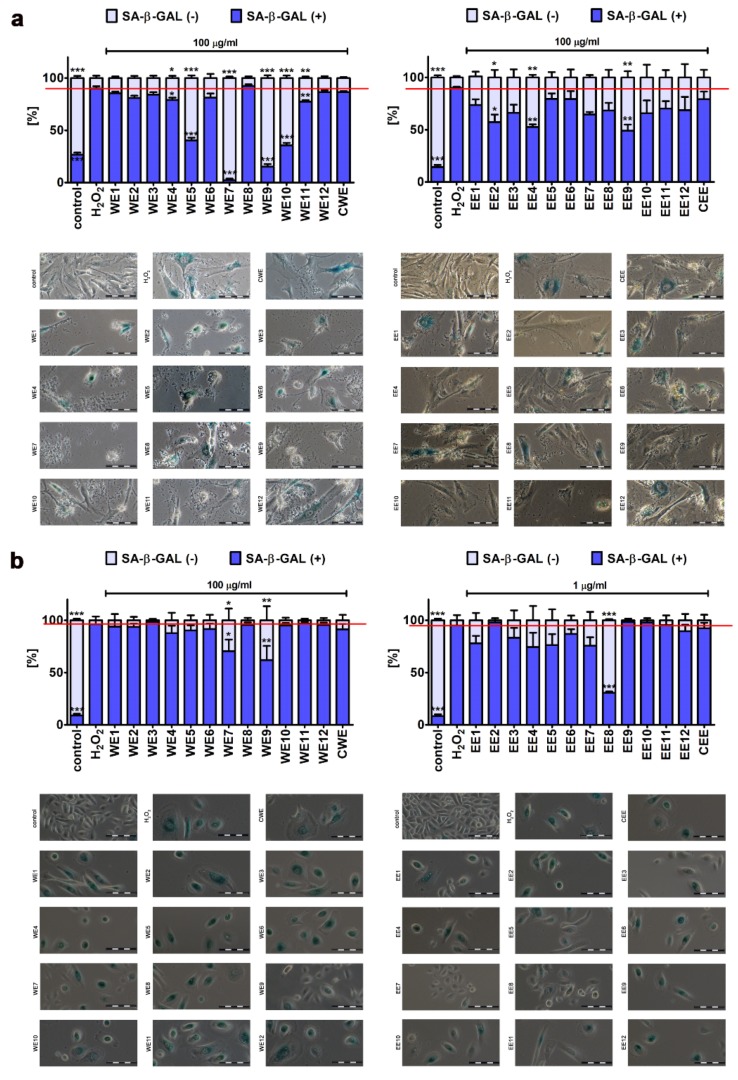
Extract-mediated changes in the levels of hydrogen peroxide-induced senescent BJ cells ((**a**), 100 µg/mL water extracts and 100 µg/mL ethanolic extracts) and HEK cells ((**b**), 100 µg/mL water extracts and 1 µg/mL ethanolic extracts). The effects of water extracts (WE, twelve modified clones from WE1 to WE12) and ethanolic extracts (EE, twelve modified clones from EE1 to EE12) are shown. Control clone water extract is denoted as CWE and control clone ethanolic extract is denoted as CEE. Senescence-associated β-galactosidase activity. Representative microphotographs are shown. Scale bars 100 μm, objective 20x. To emphasize extract action, a red horizontal line is added. Bars indicate SD, *n* = 3, *** *p* < 0.001, ** *p* < 0.01, * *p* < 0.05 compared to hydrogen peroxide treatment (ANOVA and Dunnett’s a posteriori test).

Cellular senescence may be due to telomere shortening-based limited proliferative ability of human cells in vitro (replicative senescence) or may be promoted by diverse stress stimuli, namely, physical, chemical, or biological factors (stress-induced premature senescence, SIPS) [39]. As cellular senescence may contribute to aging and age-related diseases, it has been postulated that interventions based on the suppression of induction of cellular senescence, delay the onset of cellular senescence and/or removal of senescent cells from the body may be therapeutically important [40,41]. We then analyzed if microalgal extract may promote protective effects against oxidative stress-mediated stimulation of senescence in fibroblasts and keratinocytes (Figure 9).

We used a cellular model of stress-induced premature senescence that is based on the incubation of cells with 100 µM hydrogen peroxide for 2 h, followed by a 7-day culture without an oxidant [17,19]. After treatment, about 90% population of BJ cells and 95% population of HEK cells were characterized by senescence-associated beta-galactosidase (SA-beta-gal) activity (Figure 9). We have then tested if microalgal extracts may affect the levels of SA-beta-gal-positive cells when added after hydrogen peroxide treatment (Figure 9). Interestingly, selected microalgal extracts were found to be protective against the development of oxidative stress-induced senescence in fibroblasts and keratinocytes (Figure 9). The following ranking of extract-mediated anti-senescence activity was established, namely, for the effects of water extracts in hydrogen peroxide-treated BJ and HEK cells: WE7 (2% of SA-beta-gal-positive cells) > WE9 (15% of SA-beta-gal-positive cells) > WE10 (36% of SA-beta-gal-positive cells) > WE5 (40% of SA-beta-gal-positive cells) > WE11 (78% of SA-beta-gal-positive cells) > WE4 (79% of SA-beta-gal-positive cells) and WE9 (62% of SA-beta-gal-positive cells) > WE7 (70% of SA-beta-gal-positive cells), respectively (Figure 9). Ethanolic extracts were less effective, but post-treatment with selected extracts also resulted in a decrease in the levels of SA-beta-gal-positive cells, namely EE9 (49% of SA-beta-gal-positive cells), EE4 (52% of SA-beta-gal-positive cells), and EE2 (57% of SA-beta-gal-positive cells) in hydrogen peroxide-treated BJ cells and EE8 (31% of SA-beta-gal-positive cells) in hydrogen peroxide-treated HEK cells (Figure 9). To conclude, WE7 and WE9 promoted the most pronounced anti-senescence activity in both cell lines (Figure 9). In the case of WE7, the protective effect may rely on WE7-mediated nitric oxide signaling, at least in BJ cells (Figure 8) and increased pools of niacin and biotin compared to unmodified control microalgal clone (CWE) [16]. A plethora of biological processes can be modulated by niacin-derived nucleotides (e.g., nicotinamide adenine dinucleotide, NADH, and its phosphorylated form NADPH), namely, redox reactions, antioxidative protection, DNA repair, and the activity of signaling pathways, which may stimulate some protective effects during stress conditions [42]. However, more studies are needed to establish the molecular mechanism of microalgal extract-mediated attenuation of the development of oxidative stress-induced senescence in fibroblasts and keratinocytes.

Of course, we have also tested if the treatments with microalgal extracts alone may stimulate some pro-senescence effects (Figure S3). The pro-senescence activity of microalgal extracts was limited (Figure S3). In HEK cells, only water extract WE3 promoted an increase in the levels of SA-beta-gal-positive cells compared to untreated cells, whereas treatment with ethanolic extract EE10 resulted in the most pronounced pro-senescence activity compared to other microalgal extracts in BJ cells (Figure S3).

We also observed that treatment with selected microalgal extracts for 7 days after 2 h pre-treatment with hydrogen peroxide (long-term stimulation) may result in cell loss, as judged by the analysis of microphotographs (Figure 9). As we did not reveal any signs of cytotoxicity after short-term treatments (24 h, majority of assays when cells were plated at the concentration of 10,000 cells per cm^2^) and after stimulation with microalgal extracts for 3 days (wound healing assay based on high cell density protocol; Figure S2), one can conclude that perhaps the observed decrease in cell numbers (SA-β-gal assay when cells are typically seeded at the density of 2000 cells per cm^2^ to avoid cell culture overcrowding when the test is terminated after 7 days; Figure 9) may be due to extract-mediated cytostatic effects and various cell responses based on the number of cells plated into the culture vessel. Moreover, prolonged antiproliferative effects of selected microalgal extracts after 24 h stimulation and 7 days of the removal of microalgal extracts were also noticed (Figure S3). Thus, we then decided to investigate this phenomenon more comprehensively, namely, the analysis of absolute numbers of senescent and non-senescent cells (cell count test) was performed after 2 h stimulation with hydrogen peroxide and subsequent cell culture with microalgal extracts for 7 days or 24 h treatment with microalgal extracts and subsequent cell culture without microalgal extracts for 7 days (Figure S4). In the case of 2 h pre-treatment with hydrogen peroxide followed by long-term stimulation with microalgal extracts for 7 days (Figure S4a,b), we analyzed the action of microalgal extracts compared to hydrogen peroxide treatment (Figure S4a,b) as we expected that hydrogen peroxide would promote the cytostatic effect itself. Thus, hydrogen peroxide treatment is assumed as a reference (Figure 4a,b). Cytostatic effects of microalgal extracts were limited to BJ cells treated with WE7 (a decrease in cell count of about 46% compared to hydrogen peroxide treatment, *p* < 0.001; Figure S4a). WE7-treated cells were non-senescent cells (non-stained cells; Figure 9a; WE7 treatment). In contrast, pro-stimulatory effects were observed in the case of HEK cells treated with WE9, EE7, and EE8 (an increase in cell number of about 30% compared to hydrogen peroxide treatment, *p* < 0.05, Figure S4b). These cells were also non-senescent cells (non-stained cells; Figure 9b; WE9, EE7, and EE8 treatments). More pronounced cytostatic effects of microalgal extracts were noticed when BJ cells were treated with selected ethanolic extracts for 24 h and then cultured for up to 7 days without the extracts (Figure S4c). In this case, control conditions were considered as a reference (100%; Figure S4c,d). EE1, EE3, and EE4 decreased the number of BJ cells by about 50% compared to standard growth conditions (*p* < 0.001, Figure S4c). According to microphotographs, these cells were mainly non-senescent cells (non-stained cells; Figure S3a; EE1, EE3, and EE4 treatments). Similar effects were not observed in the case of HEK cells treated with ethanolic extracts (Figure S4d). Minor to moderate cytostatic action of microalgal extracts was also noticed in the case of BJ cells treated with WE11, EE2, and EE11, and HEK cells treated with WE4 (a decrease in cell count ranging from 20% to 30% compared to the control; *p* < 0.001, *p* < 0.01 and *p* < 0.05; Figure S4c,d). Thus, one can conclude that the cytostatic action of microalgal extracts is limited, and, in some cases, extract-mediated pro-stimulatory effects are also observed (Figure S4b).

Data on *Chlorella* extract-associated anti-aging action are limited to selected models of replicative senescence, namely, human fibroblasts and myocytes at different passage numbers (young, pre-senescent, and senescent cells) [24,27,43,44,45] and UV-induced cytotoxicity and senescence [46,47]. Hot water extract of *Chlorella vulgaris* lowered the levels of DNA damage in young, pre-senescent, and senescent fibroblasts in control conditions [27] and during oxidative stress [43]. However, the antioxidant activity of hot water extract of *Chlorella vulgaris* was limited to pre-senescent fibroblasts as judged by the extract-mediated increase in catalase (CAT), superoxide dismutase (SOD), and glutathione peroxidase (GPx) activity [44]. More recently, *Chlorella* extract-mediated modulation of the expression of genes involved in oxidative stress response and DNA damage response and insulin/insulin-like growth factor-1 signaling, cell differentiation, and cell proliferation pathways was analyzed using young, pre-senescent, and senescent fibroblasts [24]. The authors concluded that *Chlorella* extract decreased the expression of *SOD1*, *CAT,* and copper chaperone *CCS* in young fibroblasts and increased the expression of *SOD2* in young, pre-senescent, and senescent fibroblasts [24]. Moreover, *Chlorella* extract promoted a decrease in mRNA levels of two regulators of cell cycle progression, namely, *TP53* (p53) and *CDKN2A* (p16) in replicative old fibroblasts that may suggest some anti-aging effects. Indeed, *Chlorella*-derived peptide (CDP) inhibited UVB-induced matrix metalloproteinase-1 (MMP-1) expression in skin fibroblasts by suppressing the expression of transcription factor AP-1 and cysteine-rich 61 (CYR61) and monocyte chemoattractant protein-1 (MCP-1) production [46]. Thus, CDP may attenuate MMP-1-stimulated UV-associated premature skin aging [46]. Similar protective effects and related mechanisms were observed against UV-induced photo-damage in *Arthrospira platensis* extract-treated human dermal fibroblasts [48].

### 3.4. Preliminary Analysis of Extract-Mediated Anticancer Activity

As *Chlorella* sp. water and ethanolic extracts have been repeatedly reported to possess pro-apoptotic and growth inhibitory activity against cancer cells of different origin both in vitro and in vivo [49,50,51], we also initially evaluated the anticancer activity of *Planktochlorella nurekis* water and ethanolic extracts (100 µg/mL) against selected cellular models of cancer in vitro, namely, MDA-MB-231 breast cancer cells, U-2 OS osteosarcoma cells, and U-251 MG glioblastoma cells (Figure S5). An MTT assay was used, and extract-mediated changes in metabolic activity of cancer cells were compared to extract-mediated changes in metabolic activity of normal human fibroblasts (BJ cells; Figure S5). Water extracts at the concentration of 100 µg/mL did not affect metabolic activity of U-2 OS and U-251 MG cells, whereas water extracts WE2, WE3, and WE5 stimulated metabolic activity of about 25% to 30% in MDA-MB-231 cells (Figure S5a; *p* < 0.05). Thus, *Planktochlorella nurekis* water extracts cannot be considered to be used in anticancer therapies, at least not those involving osteosarcoma, glioblastoma, or breast cancers (Figure S5a). In contrast, selected ethanolic extracts acted against cancer cells, especially U-2 OS and U-251 MG cells (Figure S5b). The most pronounced anticancer effects were observed when U-2 OS and U-251 MG cells were treated with EE8, EE7, EE6, and EE2 compared to similar treatments of BJ fibroblasts (Figure S5b, *p* < 0.001). However, the anticancer activity of *Planktochlorella nurekis* ethanolic extracts requires further comprehensive studies.

## 4. Conclusions

We analyzed for the first time the biological activity of twelve clones of the microalga *Planktochlorella nurekis* cells with improved biochemical features compared to their unmodified counterpart using human skin cells, namely, fibroblasts and keratinocytes, as two cellular models in vitro. As selected microalgal extracts blocked the development of oxidative stress-induced senescence in human skin cells, we strongly believe that this approach may have potential applications in such clinical contexts where protection against the occurrence of old damaged cells has great importance, e.g., in skin anti-aging therapies. Perhaps microalgal extracts may also be active against stress-induced senescence in cells derived from tissues and organs other than skin. More studies involving other cell types are needed to confirm such assumptions and reveal the underlying mechanism(s).

## Supplementary Materials

The following are available online at https://www.mdpi.com/2072-6643/12/4/1005/s1. Figure S1: Extract-mediated ERK1/2 activity in BJ cells ((a), 100 µg/mL water extracts) and HEK cells ((b), 100 µg/mL water extracts); Figure S2: Extract-mediated effects on cell migration. Scratch wound healing assay; Figure S3: Pro-senescence activity of microalgal extracts in BJ cells ((a), 100 µg/mL water extracts and 100 µg/mL ethanolic extracts) and HEK cells ((b), 100 µg/mL water extracts and 1 µg/mL ethanolic extracts); Figure S4: Extract-mediated changes in BJ (a) and HEK cell number (b) after 2 h stimulation with hydrogen peroxide and subsequent cell culture for 7 days in the presence of water (left) and ethanolic extracts (right), and the effect of 24 h treatment with water (left) and ethanolic (right) extracts on BJ (c) and HEK cell number (d) after subsequent cell culture for 7 days without microalgal extracts; Figure S5: Preliminary analysis of anticancer activity of water ((a), 100 µg/mL) and ethanolic ((b), 100 µg/mL) extracts against MDA-MB-231 breast cancer, U-2 OS osteosarcoma and U-251 MG glioblastoma cells.
