# Supplementary material for: Treatment with Modified Extracts of the Microalga Planktochlorella nurekis Attenuates the Development of Stress-Induced Senescence in Human Skin Cells
Source: Nutrients. 2020 Apr 6;12(4):1005. doi: 10.3390/nu12041005 (PMC7231398; doi:10.3390/nu12041005)

Supplementary material

Supplementary Figure 1.

Extract-mediated ERK1/2 activity in BJ cells (**a,** 100 µg/ml water extracts) and HEK cells (**b,** 100 µg/ml water extracts). The effects of water extracts (WE, twelve modified clones from WE1 to WE12) are shown. Control clone water extract is denoted as CWE. ERK1/2 activity was measured using Muse® Cell Analyzer and Muse® MAPK Activation Dual Detection Kit. Representative dot-plots are presented. Bars indicate SD, n = 3.

Supplementary Figure 2.

Extract-mediated effects on cell migration. Scratch wound healing assay. BJ cells (**a**) and HEK cells (**b**) were treated with 100 µg/ml water extracts for up to 72 h after wounding. Cell migration was evaluated under an inverted microscope. Representative microphotographs are shown. Scale bars 500 µm, objective 4x.

Supplementary Figure 3.

Pro-senescence activity of microalgal extracts in BJ cells (**a,** 100 µg/ml water extracts and 100 µg/ml ethanolic extracts) and HEK cells (**b,** 100 µg/ml water extracts and 1 µg/ml ethanolic extracts). The effects of water extracts (WE, twelve modified clones from WE1 to WE12) and ethanolic extracts (EE, twelve modified clones from EE1 to EE12) are shown. Control clone water extract is denoted as CWE and control clone ethanolic extract is denoted as CEE. Senescence-associated β-galactosidase activity. Representative microphotographs are shown. Scale bars 100 µm, objective 20x. To emphasize extract action, a red horizontal line is added. Bars indicate SD, n = 3, $^{***}p < 0.001$, $^{**}p < 0.01$, $^{*}p < 0.05$ compared to the control (ANOVA and Dunnett’s *a posteriori* test).

Supplementary Figure 4.

Extract-mediated changes in BJ (**a**) and HEK cell number (**b**) after 2 h stimulation with hydrogen peroxide and subsequent cell culture for 7 days in the presence of water (left) and

ethanolic extracts (right), and the effect of 24 h treatment with water (left) and ethanolic (right) extracts on BJ (**c**) and HEK cell number (**d**) after subsequent cell culture for 7 days without microalgal extracts. Cell number was analyzed using TC10™ automated cell counter. To emphasize extract action, a red horizontal line is added. The effects of water extracts (WE, twelve modified clones from WE1 to WE12, left) and ethanolic extracts (EE, twelve modified clones from EE1 to EE12, right) are shown. Control clone water extract is denoted as CWE and control clone ethanolic extract is denoted as CEE. Bars indicate SD, n = 3. (**a, b**) Cell number after 2 h stimulation with hydrogen peroxide is considered as 100%. $^{***}p < 0.001$, $^{*}p < 0.05$ compared to hydrogen peroxide treatment (ANOVA and Dunnett's *a posteriori* test). (**c, d**) Cell number at control growth conditions is considered as 100%. $^{***}p < 0.001$, $^{**}p < 0.01$, $^{*}p < 0.05$ compared to the control (ANOVA and Dunnett's *a posteriori* test).

Supplementary Figure 5.

Preliminary analysis of anticancer activity of water (**a,** 100 μg/ml) and ethanolic (**b,** 100 μg/ml) extracts against MDA-MB-231 breast cancer, U-2 OS osteosarcoma and U-251 MG glioblastoma cells. BJ fibroblasts were used as control normal human cells. The effects of water extracts (WE, twelve modified clones from WE1 to WE12) and ethanolic extracts (EE, twelve modified clones from EE1 to EE12) are shown. Control clone water extract is denoted as CWE and control clone ethanolic extract is denoted as CEE. To emphasize extract action, a red horizontal line is added. Extract-mediated changes in metabolic activity (MTT assay) of cancer and normal cells were investigated. Metabolic activity at standard growth conditions (control) is considered as 100%. Bars indicate SD, n = 5, $^{***}p < 0.001$, $^{**}p < 0.01$, $^{*}p < 0.05$ compared to the control (ANOVA and Dunnett's *a posteriori* test).

Supplementary Figure 1.

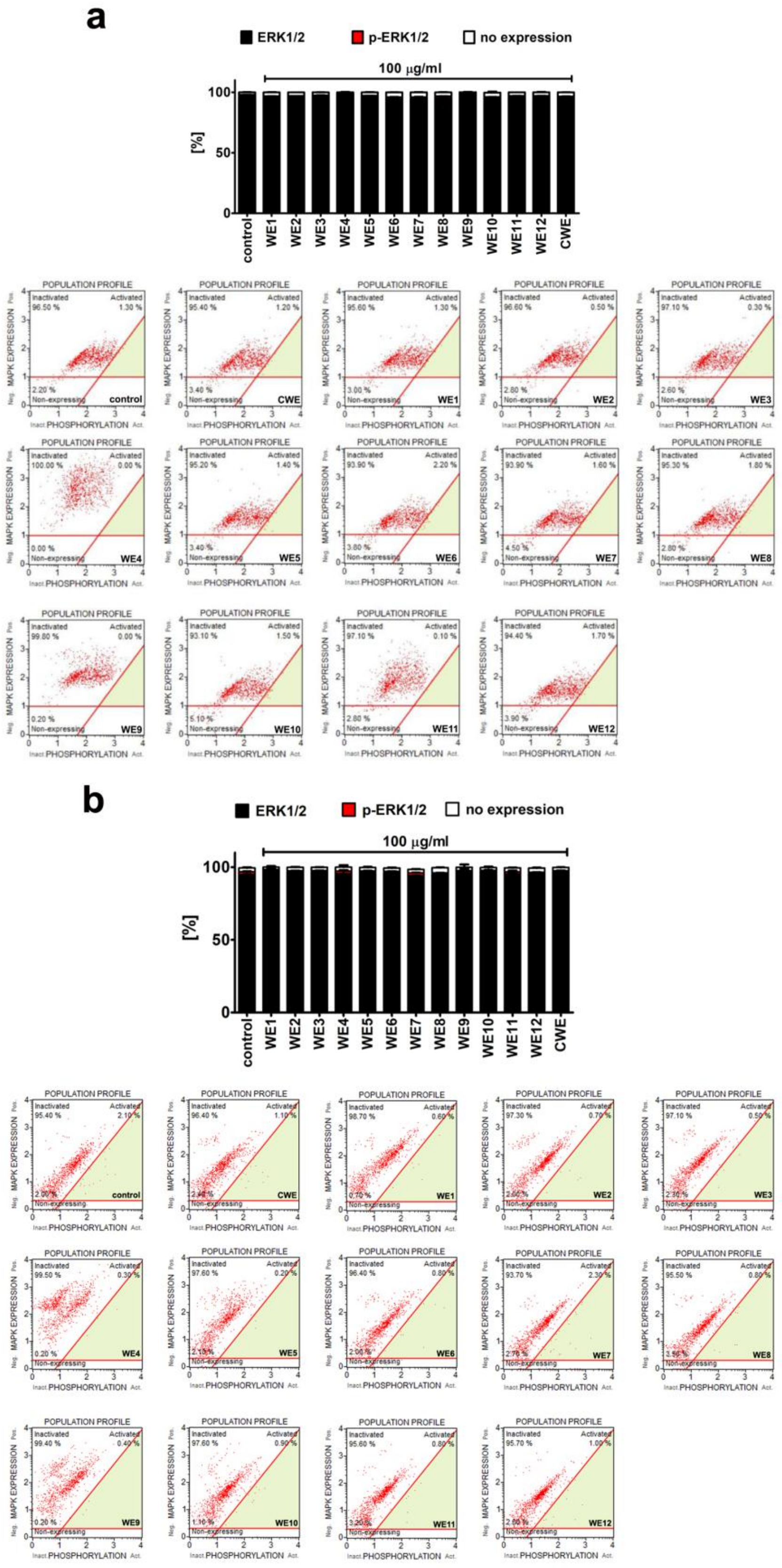

Supplementary Figure 2.

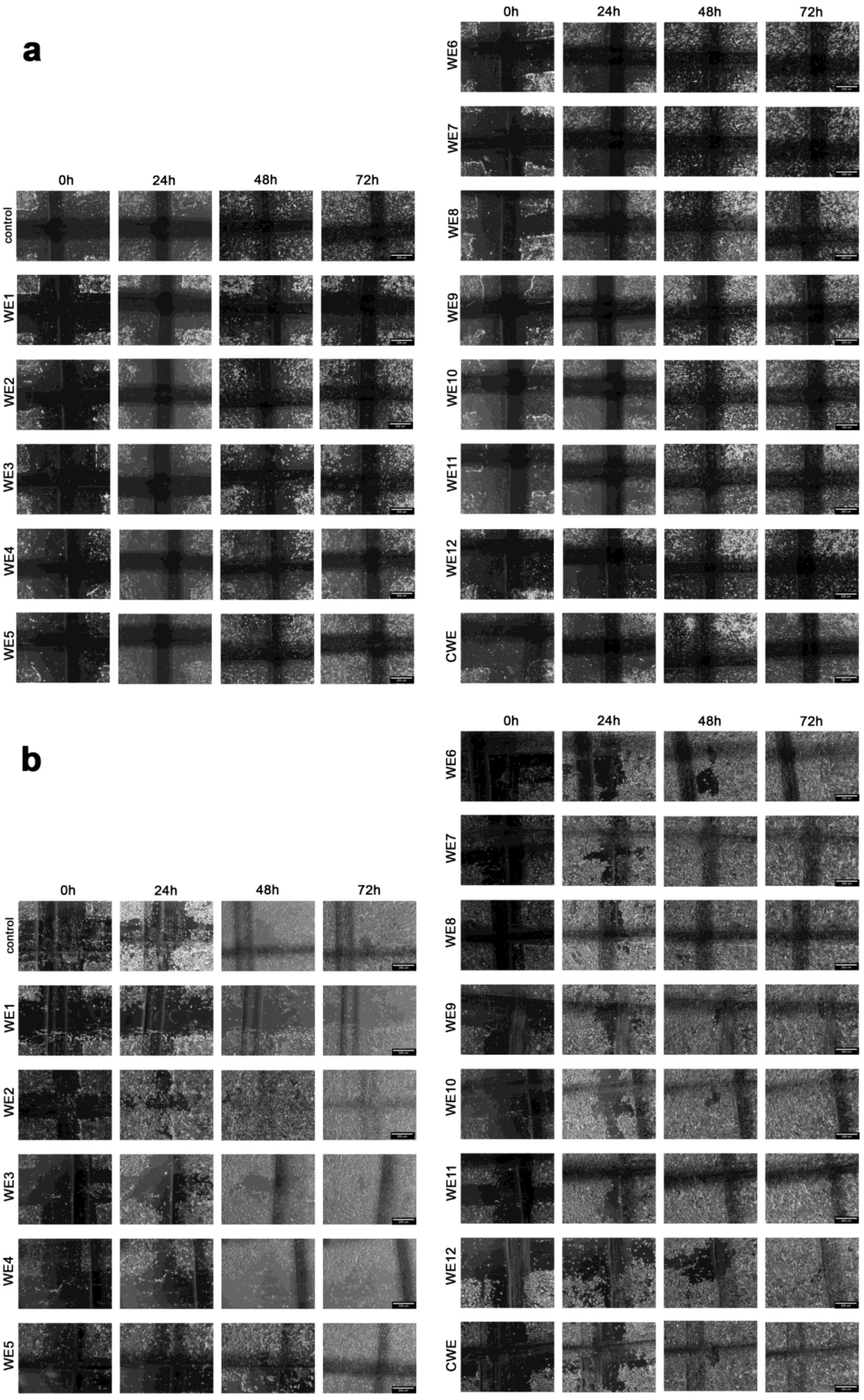

Supplementary Figure 3.

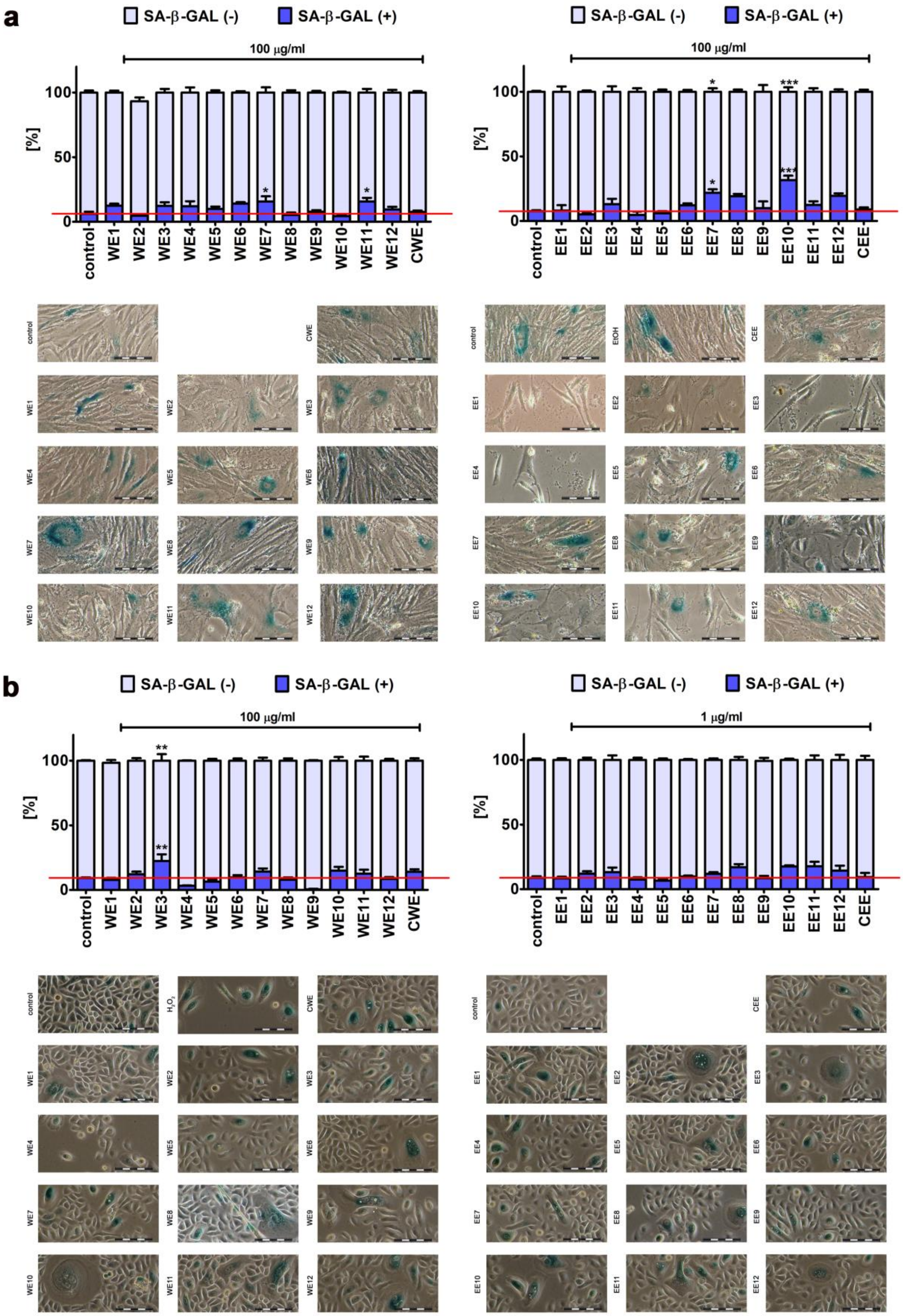

Supplementary Figure 4.

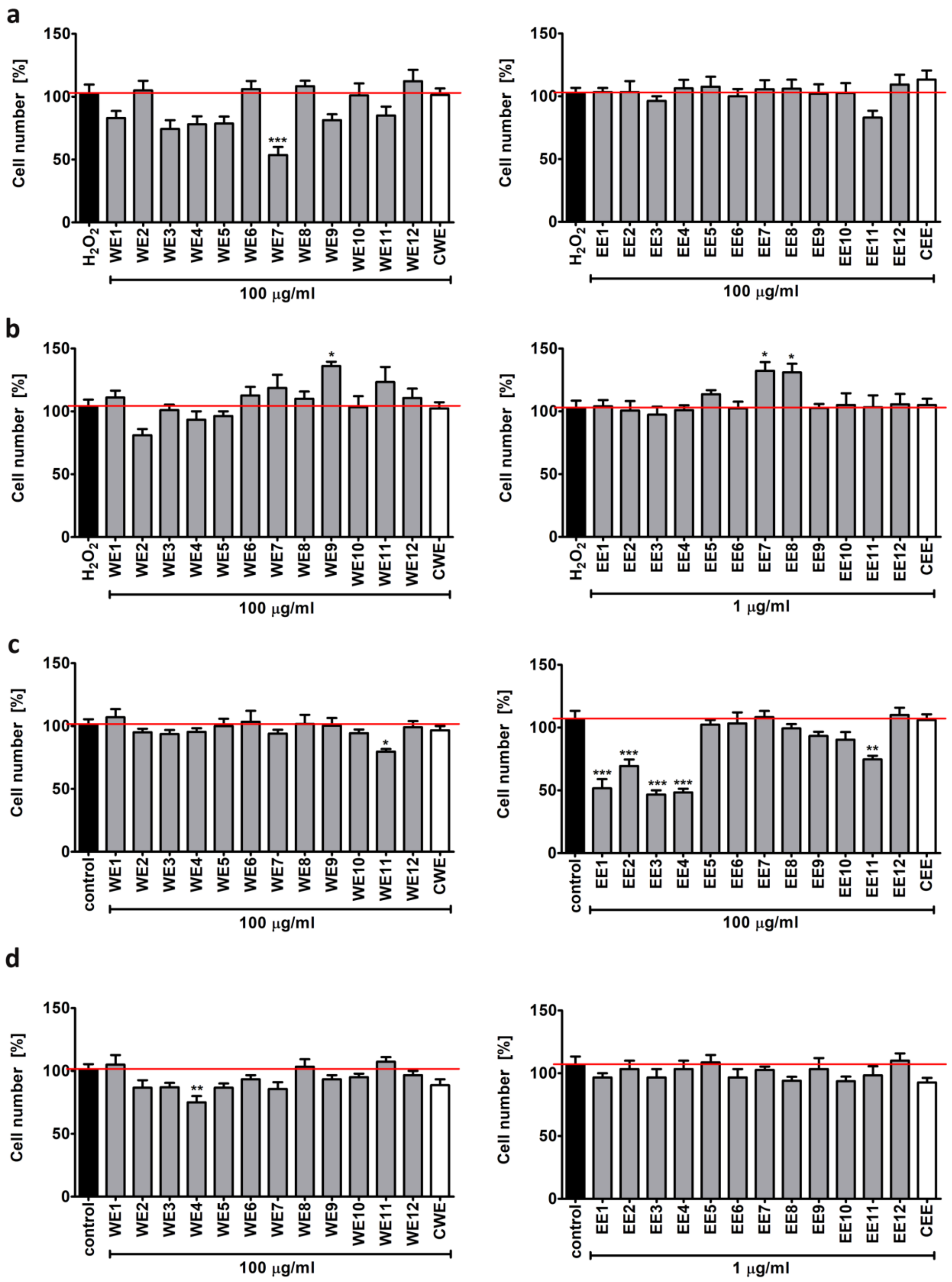

Supplementary Figure 5.

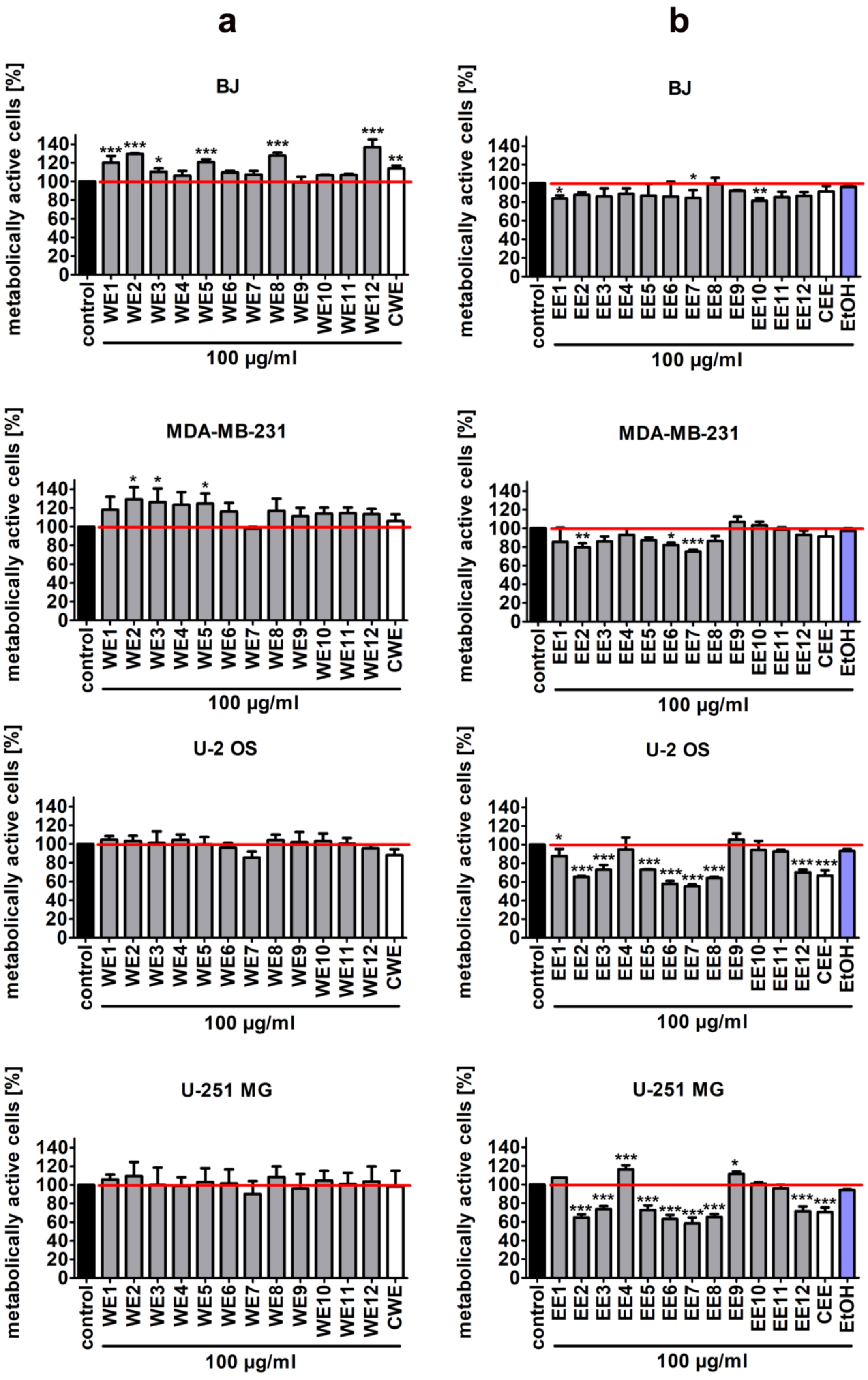

Supplement: Supplementary file 1 [file nutrients-12-01005-s001.pdf]
